# Three-dimensional digital anatomical measurements of pterygoid plates and posterior maxillary region

**DOI:** 10.1186/s12903-024-05176-8

**Published:** 2024-12-18

**Authors:** Dohyun Kwon, Soung Min Kim, Jong-Ho Lee

**Affiliations:** 1 Department of Oral and Maxillofacial Surgery, Samsung Medical Center, Sungkyunkwan University School of Medicine, Seoul, Korea; 2 Department of Oral and Maxillofacial Surgery, Dental Research Institute, School of Dentistry, Seoul National University, 101 Daehak-Ro, Jongno-Gu, Seoul, Korea; 3 Oral Oncology Clinic, National Cancer Center, Goyang, Korea; 4 Innovation Research & Support Center for Dental Science, Dental Life Science Research Institute, Seoul National University Dental Hospital, Seoul, Korea

**Keywords:** Pterygoid plate, Anatomical measurement, Transpterygoid, Maxillectomy, Infratemporal fossa, Skull base

## Abstract

**Background:**

The posterior maxilla and skull base is a region with a complex anatomy. Accurate resection of the pterygoid plate is critical during a maxillectomy procedure. However, there is a paucity of functional and anatomical studies on the pterygoid plate and skull base. This study aimed to investigate functional anatomy of the pterygoid plate and its surrounding structures in the posterior maxilla to provide a better understanding of surgical procedures in this region.

**Methods:**

3D software was used to measure 3D distances, angles, and areas of key anatomical landmarks on CT images of 100 hemifaces. Morphological classification of pterygoid plates was then performed.

**Results:**

Results of comparing right and left pterygoid plates revealed no significant differences in dimensions or angles. Comparisons between sexes revealed that a few parameters were significantly different (*P* < 0.01), including pterygoid height on the left side, distance from the zygomatico-maxillary buttress to the infraorbital fissure (Zy-IOF), and area of the left lateral pterygoid plate. The morphology of the lateral pterygoid plate was classified into four types based on the shape of the middle region: middle convex (42%), double concave (36%), flattened (10%), and middle concave (12%). The morphology of pterygoid plates was classified based on the divergence of medial and lateral pterygoid plates, with the narrow type (56%) being more common than the wide type in this study cohort.

**Conclusions:**

This 3D digital anatomical study measured key landmarks for maxillary resection. Such measurement has never been reported. This anatomical study provides surgeons with information on the anatomy of the posterior maxilla and allows for safer and more accurate resection of the difficult-to-resect posterior maxilla.

**Supplementary Information:**

The online version contains supplementary material available at 10.1186/s12903-024-05176-8.

## Background

Malignant tumor resection in the posterosuperior maxilla and skull base is a challenging surgical procedure that requires high degrees of skill and precision [1–3]. This is because of poor visualization, deep location, and the presence of critical anatomical structures. The risk of bleeding is high, which can further impair visualization.

Furthermore, the complexity of the three-dimensional anatomy of the posterosuperior maxilla and skull base often results in positive margins after surgery [4]. Local recurrence after tumor resection of the posterior part of the maxilla and maxillary sinus has been reported to occur most frequently in the posterior superior region [5]. Anatomical characteristics of the posterosuperior maxilla and skull base are also reflected in the tumor staging system of the American Joint Committee on Cancer (AJCC) 8th edition [6].

Accurate fracture of the base of the pterygoid plate is essential for en bloc resection of a tumor in the posterior superior region of the maxilla [7]. However, incomplete fracture of the pterygoid plate is frequently encountered in down fractures following maxillectomy, which often hinders en bloc tumor resection [7]. An incomplete fracture is associated with an increased risk of tumor spillage during surgery or a positive margin on final pathology [1].

Oral and maxillofacial surgeons are accustomed to performing maxillectomy via an intraoral approach. After a Le Fort I greenstick fracture, posterior pterygoid plates are further excised. However, the anatomy of the pterygoid plate area behind the posterior maxillary sinus wall is invisible to naked eyes, requiring the surgeon's attention. The width, length, and divergence between lateral and medial pterygoid plates also vary from person to person.

Anatomical knowledge of the major foramens, bone structures, and vascular structures in the skull base behind the pterygoid plate is essential. However, there is a paucity of functional anatomical studies on these structures from the perspective of oral and maxillofacial surgeons. Thus, the aim of this 3D digital anatomical study was to investigate functional anatomical relationships among main bony structures of the posterior part of the maxilla, pterygoid plates, and skull base.

## Methods

### Subjects and images

From January 2020 to February 2022, patients who visited the Department of Oral and Maxillofacial Surgery at Seoul National University Dental Hospital and underwent enhanced CT of the paranasal sinus were considered for this study. A total of 100 hemi-faces from 50 patients (25 males, 25 females) were selected based on the following inclusion and exclusion criteria:

Inclusion criteria: Patients aged 20 years or older. Patients who underwent enhanced CT of the paranasal sinus. CT scans with sufficient quality for 3D reconstruction.

Exclusion criteria: Patients with craniofacial anomalies or syndromes. Patients with a history of facial trauma or surgery. Patients with pathological conditions affecting the maxillofacial region (including fibrous dysplasia and dysgnathia).

Patients meeting the inclusion criteria were randomly selected until the target sample size was reached. This approach ensured a more consistent study population while excluding conditions that could affect the normal anatomy of the pterygoid plates and surrounding structures.

DICOM (Digital Imaging and Communications in Medicine) files of enhanced paranasal sinus CT were 3D rendered using the 3D software (Mimics® 20.0, 3-Matic® 12.0, Materialise, Leuven, Belgium). Anatomical measurements of the pterygoid plate and pterygopalatine fossa were then performed.

CT scans were acquired using a SOMATOM® Definition Edge scanner (Siemens, Erlangen, Germany). The CT exposure parameters were as follows: 120 kVp, 400 mAs, with a spiral pitch factor of 0.35, single collimation width of 0.6 mm, and total collimation width of 38.4 mm. The slice thickness was 0.6 mm, and the reconstructed slice thickness was 2 mm. All image reconstructions were performed using the syngo® via software (Siemens, Erlangen, Germany).

## Reference plane

**Fig. 1:**
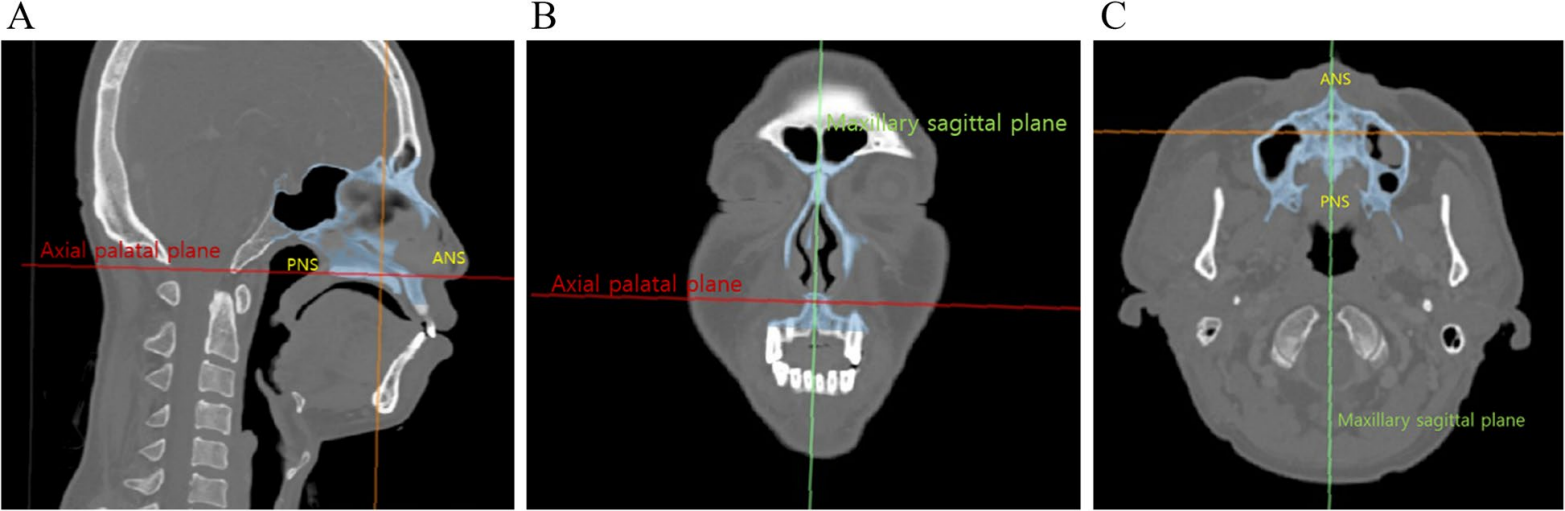
Three new reference planes were established for the study. **a** The axial palatal plane, **b** maxillary sagittal plane, and **c** coronal plane

Three new reference planes, the axial palatal plane, maxillary sagittal plane, and coronal plane, were established for this study. They were set as follows (Fig. 1).

- Axial palatal plane: perpendicular to the maxillary sagittal plane and passing through the ANS and PNS
- Maxillary sagittal plane: plane passing through the anterior nasal spine (ANS), posterior nasal spine (PNS), and nasion (N)
- Coronal plane: perpendicular to the axial palatal plane and maxillary sagittal plane

### Computed tomography and 3D model analysis

Oral and maxillofacial surgeons with more than 10 years of experience performed anatomical measurements through 3D models. This retrospective study was exempted from approval from the Institutional Review Board of the SNUDH (Seoul National University Dental Hospital) (SNUDH IRB No. ERI22002).

Anatomical measurements were performed using both Mimics® 20.0 for 3D model creation and segmentation, and 3-matic® 12.0 for detailed 3D measurements and surface area calculations. The following measurements were obtained (Table 1).

**Table 1:** Measurement Types and Descriptions for Three-dimensional Anatomical Analysis

Measurement Type	Measurement Description	Reference Figure
**Linear measurements**		
AP Length (Superior)	From the most anterior part of the concavity of the greater wing of the sphenoid bone to the anterior foramen ovale (upper 1/3)	Figure 2
AP Length (Middle)	From the most anterior point of the lateral pterygoid plate to the most convex area near the center (middle 1/3)	Figure 2
AP Length (Inferior)	From the lowest part of the pterygomaxillary junction to the most convex point of the inferior edge of the lateral pterygoid plate	Figure 2
MSLM	From the lateral part of the piriform aperture to the anterior of the descending palatine canal at the lowest level of the inferior nasal concha	Figure 3a
MSLL	From the anterolateral angle to the posterolateral angle of the maxillary sinus on axial CT image	Figure 3a
Height of Pterygoid Plate	From the lowest point of the pterygomaxillary junction to the base of the lateral pterygoid plate	Figure 2
Zy to Infraorbital Fissure	Distance from the most concave point of the zygomatico-maxillary buttress to the most lateral point of the infraorbital fissure	Figure 3b
Pterygomaxillary Junction to Foramen Ovale	Distance from the lowest point of the pterygomaxillary junction to the foramen ovale	Figure 3b
Infraorbital Rim to Foramen Ovale	Distance from the infraorbital rim to the most anterior-inferior point of the foramen ovale	Figure 3b
ICA to Lateral Pterygoid Plate	Distance from the internal carotid artery to the lateral pterygoid plate on axial CT image	Figure 4a
ICA to Medial Pterygoid Plate	Distance from the internal carotid artery to the medial pterygoid plate on axial CT image	Figure 4b
**Angular Measurements**		
Divergence Angle	Angle between lines connecting the posterior point of the greater palatine canal, vertex of the lateral pterygoid plate, and vertex of the medial pterygoid plate on axial CT image	Figure 3a
AMA	Anterior maxillary wall angulation; angle between MSLM and MSLL on axial CT image at the level of the inferior nasal concha	Figure 3a
**Surface Area**		
Lateral Pterygoid Plate	Measured using the polygon mark tool in 3-Matic® software, carefully selecting the outline of the lateral pterygoid plate	Figure 5a, b

#### Linear Measurements

**Fig. 2:**
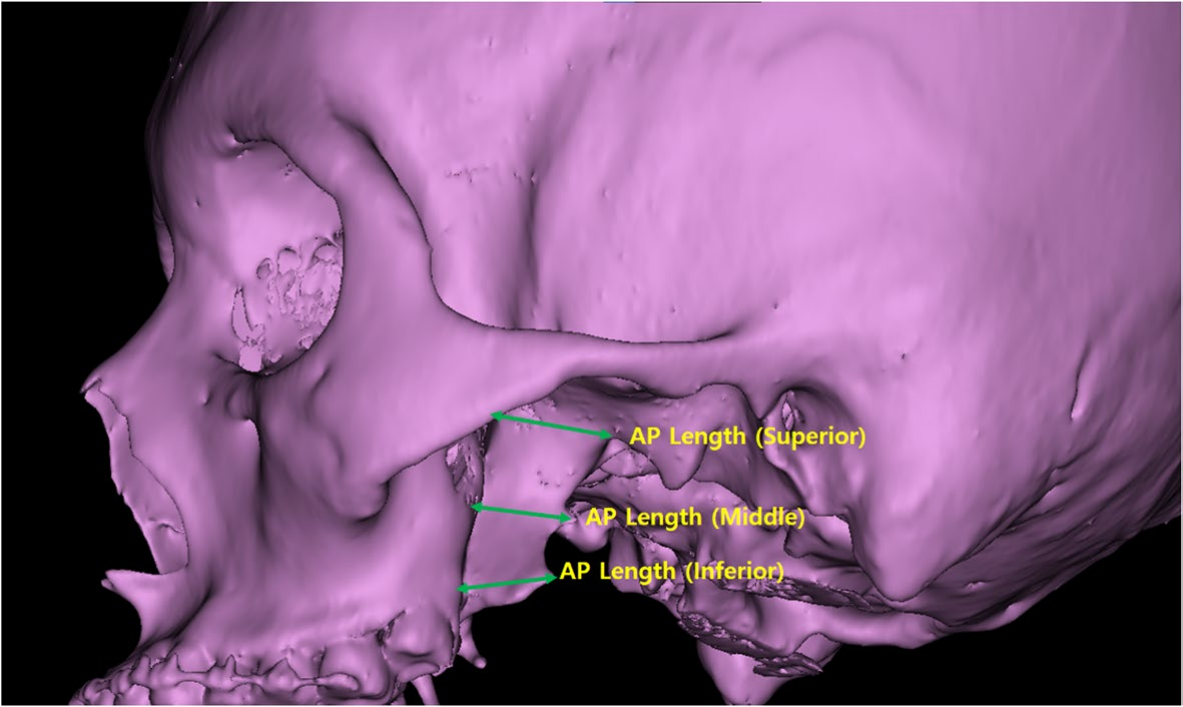
Anteroposterior (AP) length measurements of the lateral pterygoid plate at three levels: Superior: From anterior concavity of greater wing of sphenoid to anterior foramen ovale. Middle: From anterior point at inferior nasal concha level to central convex area. Inferior: From lowest pterygomaxillary junction to convex point on inferior edge

**Fig. 3:**
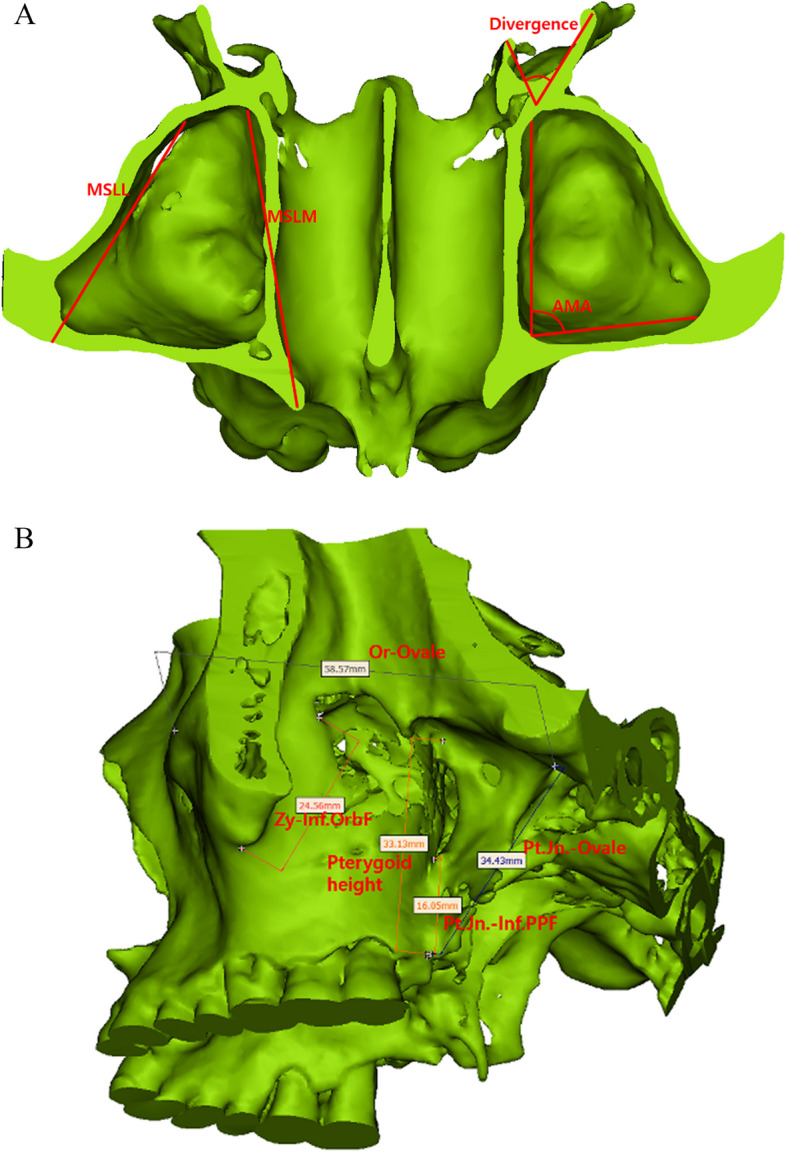
**A** Maxillary sinus dimensions of medial and lateral wall and divergence between medial and lateral pterygoid plate. **B** Various important anatomical measurement for maxillectomy and pterygopalatine fossa area surgery were measured

**Fig. 4:**
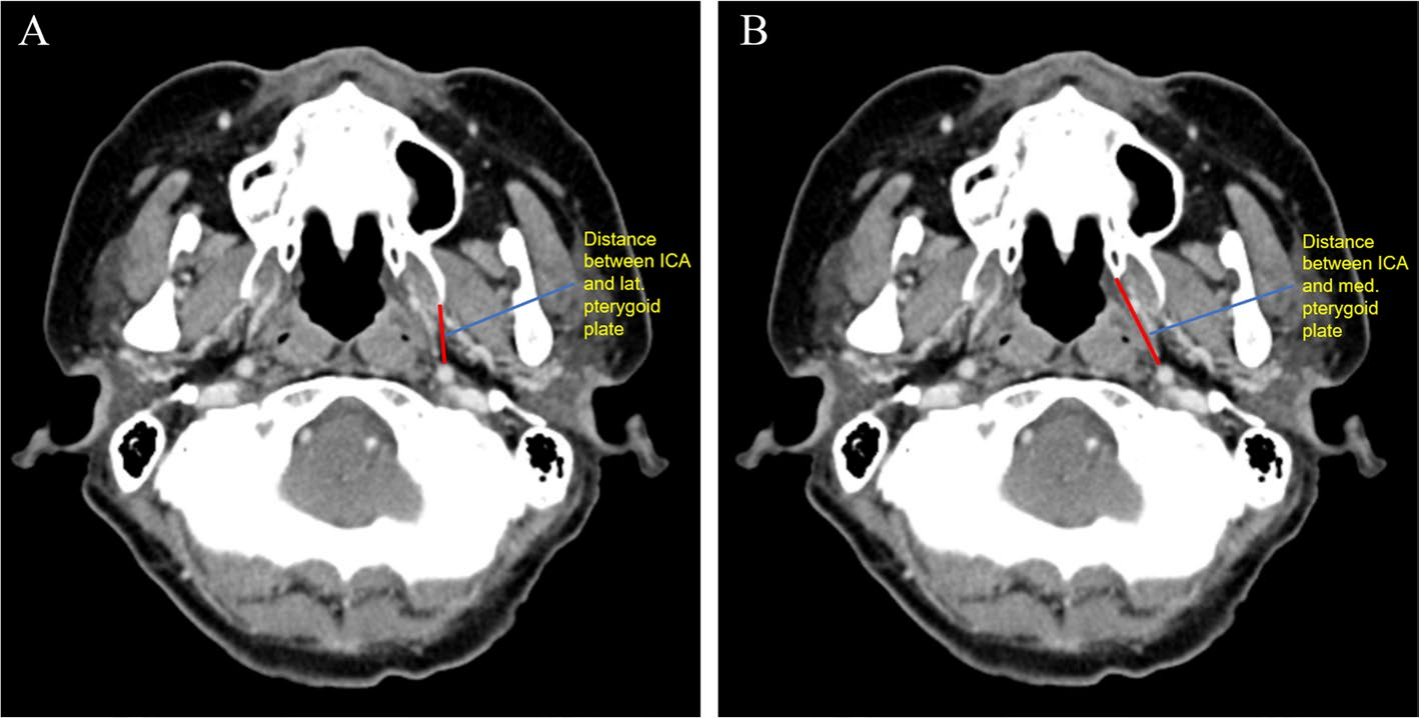
The distance from the internal carotid artery (ICA) to the (**A**) lateral and (**B**) medial pterygoid plates was measured on the axial CT image at the level of nasal floor

1. Anteroposterior (AP) Length of the Lateral Pterygoid Plate:
  A. Superior Level (Upper 1/3): Measured from the most anterior part of the concavity of the greater wing of the sphenoid bone to the anterior foramen ovale (Fig. 2).
  B. Middle Level (Middle 1/3): Measured from the most anterior point of the lateral pterygoid plate at the inferior nasal concha level to the most convex area near the center (Fig. 2).
  C. Inferior Level (Lower 1/3): Measured from the lowest part of the pterygomaxillary junction to the most convex point of the inferior edge of the lateral pterygoid plate (Fig. 2).
2. Maxillary Sinus Length:
  A. Medial (MSLM, Maxillary Sinus Length Medial): From the lateral part of the piriform aperture to the anterior of the descending palatine canal on the axial CT image at the lowest level of the inferior nasal concha (Fig. 3a).
  B. Lateral (MSLL, Maxillary Sinus Length Lateral): From the anterolateral angle to the posterolateral angle of the maxillary sinus on the axial CT image (Fig. 3a).
3. Height of the Pterygoid Plate: Measured from the lowest point of the pterygomaxillary junction to the base of the lateral pterygoid plate (Fig. 2).
4. Other Linear Measurements:
  A. Zygomatico-maxillary buttress (Zy) to Infraorbital Fissure: From the most concave point of the zygomatico-maxillary buttress to the most lateral point of the infraorbital fissure (Fig. 3b).
  B. Pterygomaxillary Junction to Foramen Ovale: From the lowest point of the pterygomaxillary junction to the foramen ovale (Fig. 3b).
  C. Infraorbital Rim to Foramen Ovale: From the infraorbital rim to the most anterior-inferior point of the foramen ovale (Fig. 3b).
  D. Internal Carotid Artery (ICA) to Lateral and Medial Pterygoid Plates: Measured on axial CT images at the level of the nasal floor (Fig. 4a, b).

#### Angular measurements

1. Divergence Angle: Measured between lines connecting the posterior point of the greater palatine canal, the vertex of the lateral pterygoid plate, and the vertex of the medial pterygoid plate on axial CT images (Fig. 3a).
2. Anterior Maxillary Wall Angulation (AMA): The angle between MSLM and MSLL on axial CT images at the level of the inferior nasal concha (Fig. 3a).

##### Surface area measurement

**Fig. 5:**
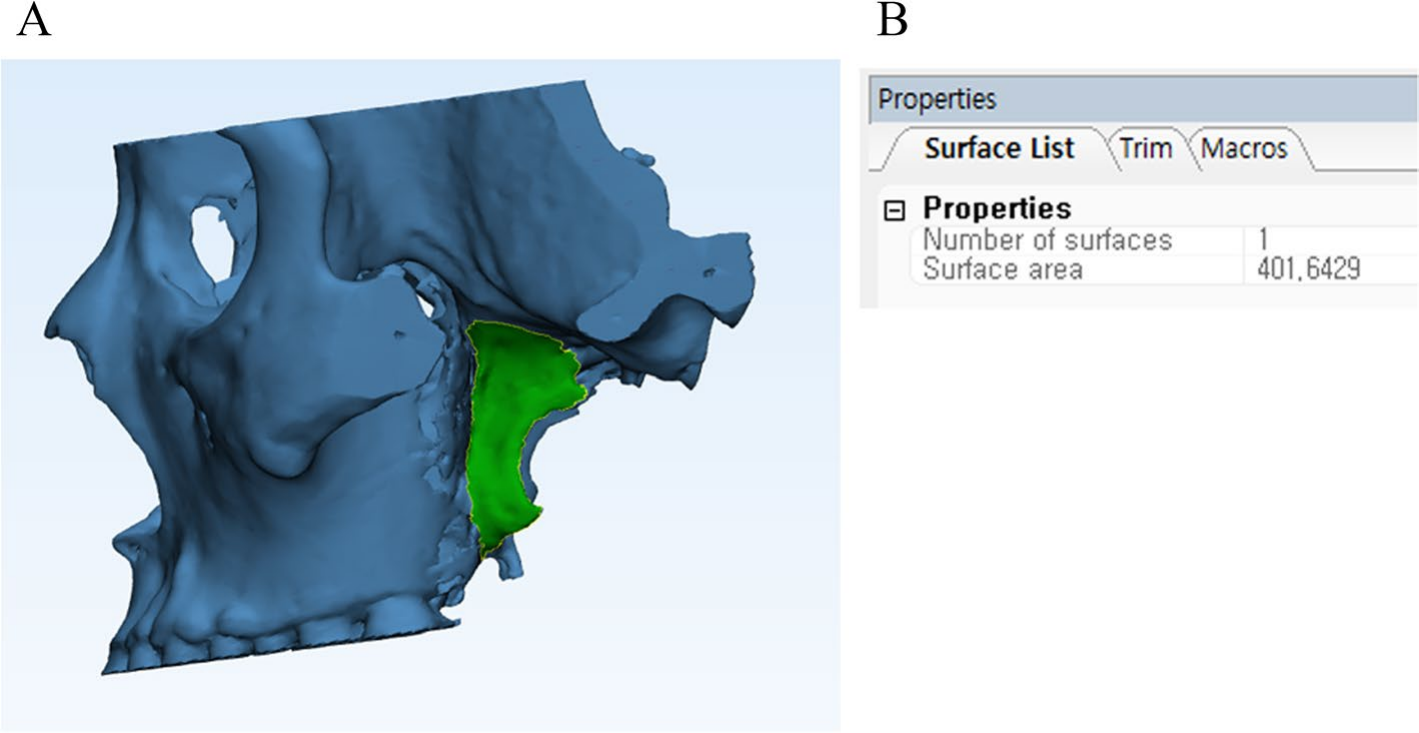
**A** Surface area of lateral pterygoid plate, **B** The program function automatically calculates the selected surface area

The surface area of the lateral pterygoid plate was measured using the polygon mark tool in 3-Matic® software by carefully selecting the outline of the lateral pterygoid plate (Fig. 5a, b).

### Classification of the morphology of the lateral pterygoid plate

The lateral pterygoid plate was segmented and analyzed using Mimics® software (Mimics® 20.0, 3-Matic® 12.0, Materialise, Leuven, Belgium) based on CT Digital Imaging and Communications in Medicine (DICOM) data reconstructed into a 3D skull model. The classification of the lateral pterygoid plate was performed based on the following criteria:

1. Segmentation and Delineation: The lateral pterygoid plate was segmented from the base of the skull to its lower border, and its morphology was analyzed.
2. Division into Thirds: The plate was divided into three equal parts (upper, middle, and lower thirds) to facilitate detailed morphological assessment.
3. Morphological Classification

**Fig. 6:**
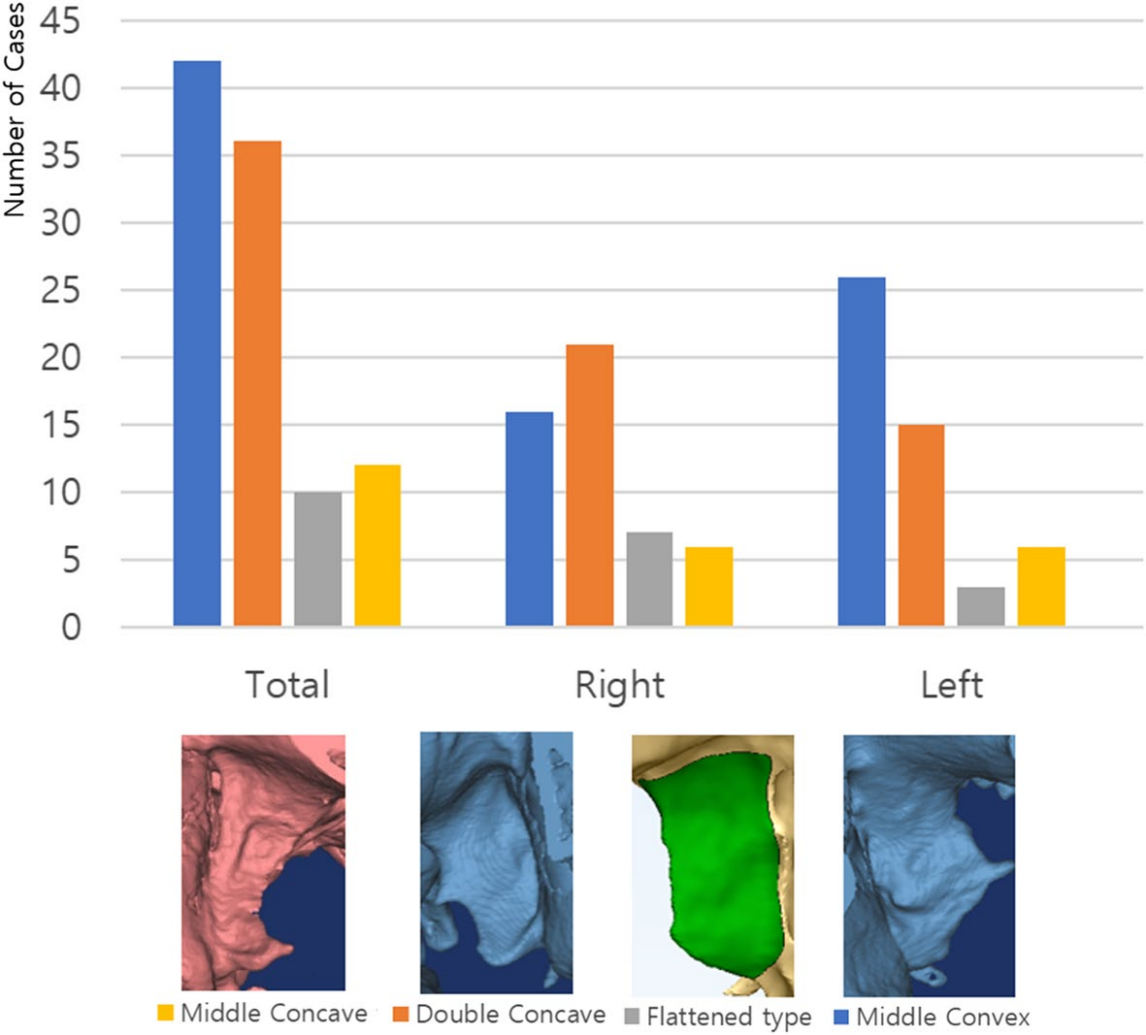
Graphic representation of the shape and distribution of the lateral pterygoid plate

The pterygoid plates were classified into four distinct types: Middle Convex type, Double Concave type, Flattened type, and Middle Concave type. These types are visually illustrated by the small 3D images at the bottom of Fig. 6, which correspond to the morphological differences discussed here.

A. Flattened Type: The plate appears predominantly flat throughout its entire length, without significant visible convexity or concavity in any of the three sections (upper, middle, lower).
B. Middle Convex Type: The middle third of the plate exhibits a noticeable outward curvature. The upper and lower thirds show a gradual slope towards this convex middle section, creating a smooth transition.
C. Double Concave Type: The middle third of the plate is visibly convex, while the upper and lower thirds display clear inward curvatures. This results in a distinctive S-shaped profile when viewed laterally.
D. Middle Concave Type: The middle third of the plate shows a visible inward curvature, while the upper and lower thirds appear relatively straight or slightly curved outward.

4. Standardization Protocol: To ensure consistency and reduce subjectivity, a standardized orientation protocol was established to align all 3D models consistently before assessment, and reference images for each type were created and used as visual guides during classification.

### Classification according to the degree of divergence of the pterygoid plate

The divergence between the medial and lateral pterygoid plates was classified into two categories: wide and narrow. This classification was based on the measured divergence angle between the plates. The median value from the measured data, 58°, was used as the critical threshold for this classification. Divergence angles greater than 58° were classified as wide, and those less than 58° were classified as narrow. This threshold ensures that the classification is data-driven and accurately reflects the distribution of divergence within the study population.

### Verification of measurement error (intraexaminer reliability)

Reproducibility of the measurement error was determined by remeasuring 10 images randomly selected by the same examiner at intervals of one week. The reproducibility of the measurements was calculated using Dahlberg's formula [8]. The Dahlberg error (measurement error; ME), D, was defined as follows:

$$\text{D }=\sqrt{\sum_{i=1}^{N}\frac{{d}_{i}^{2}}{2N}}$$

Relative measurement error (RME) or relative Dahlberg error (RDE) was calculated using the following formula:

$$\text{RME }(\text{percent})= \frac{D}{\left(\textit{mean of two corresponding measurements}\right)}\times 100$$

### Statistical analysis

Means and standard deviations (SDs) of statistical variables were calculated. A normality test of paired data was first performed. If data were normally distributed, paired t tests or independent t tests were used. Otherwise, the Wilcoxon signed rank test was used.

The difference between left and right sides was evaluated using paired t tests. Differences between men and women were evaluated using independent t tests.

All statistical analysis was performed using SPSS software version 26(SPSS Inc., Chicago, IL, USA). A *P*-value of less than 0.01 was considered significant.

## Results

### Repeatability of measurements (intra-examiner reliability)

Intra-examiner reliability was expressed as measurement error (ME) calculated from Dahlberg’s formula. Relative measurement error (RME), the limit of agreement (LoA), and the intraclass coefficient (ICC) were calculated.

Dahlberg’s error showed sufficiently small error values, ranging from 0.61 mm to 1.64 mm for linear variables, 2.14° – 3.37° for angular variables, and 30.93 mm^2^ to 32.93mm^2^ for area variables. The smallest relative measurement error was 2.22% for MSLM, while the largest was 10.91% for the mid-1/3 length. Intraclass correlation coefficients ranged from 80 to 100%, indicating high reproducibility.

### 3D anatomical measurements – Pterygoid plate, maxillary sinus, and maxilla

Anatomical measurements of the pterygoid plates and surrounding structures revealed considerable variation in dimensions and spatial relationships. These measurements were analyzed for potential differences between left and right sides using paired t-tests when normality was satisfied, and Wilcoxon signed-rank tests otherwise.

The lateral pterygoid plate exhibited varying lengths across its vertical extent. In the superior region, the mean length was 17.62 ± 2.4 mm on the right side and 17.70 ± 2.49 mm on the left side. The middle portion showed dimensions of 13.38 ± 3.38 mm on the right and 13.04 ± 3.39 mm on the left. The inferior segment demonstrated lengths of 13.38 ± 4.03 mm on the right and 14.06 ± 4.19 mm on the left.

Divergence between the lateral and medial pterygoid plates was 58.64 ± 12.62° on the right and 57.99 ± 12.23° on the left. The distance from the medial pterygoid plate to the internal carotid artery was 28.37 ± 3.50 mm on the right and 27.92 ± 3.14 mm on the left, while the lateral pterygoid plate was situated 20.85 ± 4.86 mm on the right and 20.85 ± 4.83 mm on the left from the same artery. The pterygoid junction's proximity to the inferior pterygopalatine fossa was 18.95 ± 2.58 mm on the right and 19.16 ± 2.40 mm on the left.

Pterygoid height was 28.50 ± 2.41 mm on the right and 28.76 ± 2.67 mm on the left. The distance from the pterygoid junction to the foramen ovale was 30.86 ± 2.17 mm on the right and 30.79 ± 3.71 mm on the left. Lastly, the surface area of the lateral pterygoid plate was 494.15 ± 103.64 mm^2^ on the right and 493.61 ± 89.82 mm^2^ on the left (Table 2).

**Table 2:** 3D anatomical measurements – Pterygoid plate

Parameter	Mean ± SD		Comparison (Right vs Left)		
	**Right (*n* = 50)**	**Left (*n* = 50)**	**Statistical value**	***p* value**	**95% CI (Lower, Upper)**
Pterygoid_Superior (mm)	17.62 ± 2.4	17.70 ± 2.49	0.343^a^	0.732	(16.89, 18.35)
Pterygoid_Middle (mm)	13.38 ± 3.38	13.04 ± 3.39	1.027^b^	0.31	(12.42, 14.34)
Pterygoid_Inferior (mm)	13.38 ± 4.03	14.06 ± 4.19	0.010^a^	0.992	(12.20, 14.56)
ICA_mPt (mm)	28.37 ± 3.50	27.92 ± 3.14	2.337^b^	0.024	(27.42, 29.32)
ICA_lPt (mm)	20.85 ± 4.86	20.85 ± 4.83	0.009^b^	0.993	(19.56, 22.14)
Pt.Jn._Inf.PPF (mm)	18.95 ± 2.58	19.16 ± 2.40	0.814^b^	0.42	(18.28, 19.62)
Pterygoid_height (mm)	28.50 ± 2.41	28.76 ± 2.67	1.124^b^	0.266	(27.89, 29.11)
Pt.Jn_Ovale (mm)^a^	30.86 ± 2.17	30.79 ± 3.71	1.245^b^	0.213	(30.33, 31.39)
Lateral pterygoid area (mm^2^)	494.15 ± 103.64	493.61 ± 89.82	0.048^b^	0.962	(462.85, 525.45)
Divergence(°)	58.64 ± 12.62	57.99 ± 12.23	0.058^a^	0.954	(55.29, 61.99)

Statistical value: z-statistic (^a^Wilcoxon signed-rank test) or t-statistic (^b^paired t test)

Various measurements of the maxillary sinus and maxilla were analyzed for potential differences between left and right sides. The length of the medial maxillary sinus was 37.94 ± 3.74 mm on the right and 38.54 ± 4.37 mm on the left, while the lateral maxillary sinus length was 33.24 ± 4.28 mm on the right and 32.51 ± 4.31 mm on the left. The maxillary sinus anterior angle was 64.52 ± 8.16° on the right and 64.14 ± 7.92° on the left.

Distances between specific anatomical landmarks were also measured. The distance between the Orbitale and foramen ovale was 57.07 ± 3.89 mm on the right and 56.86 ± 3.65 mm on the left. The distance between the Zy (zygomatico-maxillary buttress most concave point) and the inferior orbital fissure was 26.60 ± 1.83 mm on the right and 26.77 ± 1.96 mm on the left.

Statistical analysis revealed no significant differences in any of these measurements between the left and right sides (Table 3).

**Table 3:** 3D anatomical measurements of the maxillary sinus and maxilla

Parameter	Mean ± SD		Comparison (Right vs Left)		
	**Right (*n* = 50)**	**Left (*n* = 50)**	**Statistical value**	***p* value**	**95% CI (Lower, Upper)**
MSLM (mm)	37.94 ± 3.74	38.54 ± 4.37	1.68^a^	0.099	(36.95, 38.93)
MSLL (mm)	33.24 ± 4.28	32.51 ± 4.31	1.718^a^	0.092	(32.08, 34.40)
AMA (°)	64.52 ± 8.16	64.14 ± 7.92	0.395^a^	0.695	(62.28, 66.76)
Or_Ovale (mm)	57.07 ± 3.89	56.86 ± 3.65	1.266^a^	0.212	(56.00, 58.14)
Zy_IOF (mm)	26.60 ± 1.83	26.77 ± 1.96	1.597^a^	0.266	(26.07, 27.13)

statistical value: t-statistic (^a^paired t test)

### Comparison of parameters between sexes

Anatomical measurements were compared between men and women using appropriate statistical tests. The independent t-test was employed when the data satisfied normality assumptions, while the Mann–Whitney test was used for non-normally distributed data.

Analysis revealed statistically significant differences (*p* < 0.01) between sexes in three specific measurements:

- 1) Pterygoid height on the left side: The mean height in males was 29.76 ± 2.99 mm (95% CI: 28.51–31.01 mm) compared to 27.75 ± 1.88 mm (95% CI: 26.97–28.53 mm) in females, with a mean difference of 2.01 mm (95% CI: 0.70–3.32 mm). 2) Zy-IOF distance on the right side: Males had a mean distance of 27.26 ± 1.86 mm (95% CI: 26.48–28.04 mm) compared to 25.94 ± 1.58 mm (95% CI: 25.28–26.60 mm) in females, with a mean difference of 1.32 mm (95% CI: 0.39–2.25 mm). 3) Lateral pterygoid surface area on the left side: The mean area in males was 531.56 ± 72.81 mm2 (95% CI: 501.48–561.64 mm2) compared to 455.65 ± 90.39 mm2 (95% CI: 418.13–493.17 mm2) in females, with a mean difference of 75.91 mm2 (95% CI: 29.95–121.87 mm2). All other anatomical parameters examined showed no statistically significant differences between men and women (Table 4).

**Table 4:** Comparison of parameters between sexes

Parameter	Right (Mean ± SD)				Left (Mean ± SD)			
	**Male (*n* = 50)**	**Female (*n* = 50)**	**Statistical value**	***p* value**	**Male (*n* = 50)**	**Female (*n* = 50)**	**Statistical value**	***p* value**
Pterygoid_Superior (mm)	17.82 ± 2.73 (16.69–18.95)	17.41 ± 2.15	0.589^a^	0.503	17.69 ± 3.01	17.71 ± 1.90	−0.019^a^	0.58
Pterygoid_middle (mm)	13.67 ± 3.25 (12.32–15.02)	13.09 ± 3.55	0.598^b^	0.553	13.49 ± 3.57	12.60 ± 3.21	0.923^b^	0.361
Pterygoid_Inferior (mm)	14.10 ± 4.33	13.03 ± 3.72	0.939^b^	0.352	15.11 ± 4.38	13.01 ± 3.78	1.813^a^	0.16
Divergence (°)	59.81 ± 12.02	57.48 ± 13.33	0.65^b^	0.904	58.68 ± 11.76	57.30 ± 12.90	0.393^a^	0.516
MSLM (mm)	38.87 ± 3.40	37.00 ± 3.88	1.806^b^	0.077	39.93 ± 3.28	37.14 ± 4.91	2.367^b^	0.015
MSLL (mm)	34.27 ± 4.28	32.20 ± 4.12	1.74^b^	0.088	33.40 ± 4.09	31.62 ± 4.43	1.475^b^	0.147
AMA (°)	66.19 ± 7.40	62.85 ± 8.68	1.466^b^	0.149	66.51 ± 8.04	61.77 ± 7.20	2.196^b^	0.033
ICA_mPt (mm)	29.32 ± 2.87	27.41 ± 3.86	1.987^b^	0.053	28.45 ± 3.11	27.38 ± 3.14	1.211^a^	0.265
ICA_lPt (mm)	21.21 ± 5.51	20.48 ± 4.17	0.522^b^	0.604	20.90 ± 5.27	20.80 ± 4.46	0.071^b^	0.944
Or_Ovale (mm)	57.76 ± 3.66	56.37 ± 4.06	1.274^b^	0.209	57.47 ± 3.36	56.25 ± 3.90	1.189^b^	0.24
Pt.Jn_inf.PPF (mm)	18.60 ± 2.87	19.31 ± 2.25	0.98^b^	0.332	19.28 ± 2.53	19.03 ± 2.30	0.37^b^	0.713
Pterygoid height (mm)	29.33 ± 2.52	27.66 ± 2.02	2.585^b^	0.013	29.76 ± 2.99	27.75 ± 1.88	2.846^b^	< 0.01
Pt._Jn._Ovale (mm)	31.44 ± 2.28	30.28 ± 1.94	1.926^a^	0.09	31.92 ± 2.66	29.65 ± 4.28	2.247^b^	0.029
zy_IOF (mm)	27.26 ± 1.86	25.94 ± 1.58	2.714^b^	< 0.01	27.57 ± 1.92	25.98 ± 1.68	3.136^a^	0.031
Lateral_pterygoid_area (mm^2^)	525.68 ± 86.06	462.63 ± 111.58	2.237^b^	0.03	531.56 ± 72.81	455.65 ± 90.39	3.27^b^	< 0.01

Statistical value: z-statistic (^a^Mann-Whitney U test) or t-statistic (^b^independent t-test)

### Classification of pterygoid plate morphology

The morphology of the lateral pterygoid plate was classified into four types based on the shape of the middle region: middle convex, double concave, flattened, and middle concave. On the right side, the double concave type was the most frequent (42%), followed by the middle convex type (32%). The flattened type accounted for 14%, and the middle concave type accounted for 12%. On the left side, the middle convex type was the most frequent (52%), followed by the double concave type (30%). The middle concave type constituted 12%, and the flattened type constituted only 6% (Fig. 6, Table 5).

**Table 5:** Distribution according to morphological classification of lateral pterygoid plate

	**Total**	**Right**	**Left**
Middle convex type	42	16	26
Double concave type	36	21	15
Flattened type	10	7	3
Middle concave type	12	6	6
	100	50	50

We conducted an additional analysis to assess the symmetry of pterygoid plate morphology between left and right sides. Out of the 50 individuals examined, 31 (62%) exhibited symmetrical morphology, with the same type of pterygoid plate on both sides. The remaining 19 individuals (38%) showed asymmetrical morphology. Among the symmetrical cases, the double concave type was most common (22% of all individuals), followed closely by the middle convex type (20%). In asymmetrical cases, the most frequent combination was double concave on one side and middle convex on the other (16% of all individuals). There was a slight tendency towards more symmetry in males (68% symmetrical) compared to females (56% symmetrical), though this difference was not statistically significant (chi-square test, *p* = 0.382).

### Classification according to the degree of divergence of the pterygoid plate

The morphology of pterygoid plates was classified based on the divergence of the medial and lateral pterygoid plates. The median value of divergence (58°) was set as the critical value. Values larger than this threshold were classified as wide and values smaller than this threshold were classified as narrow.

**Fig. 7:**
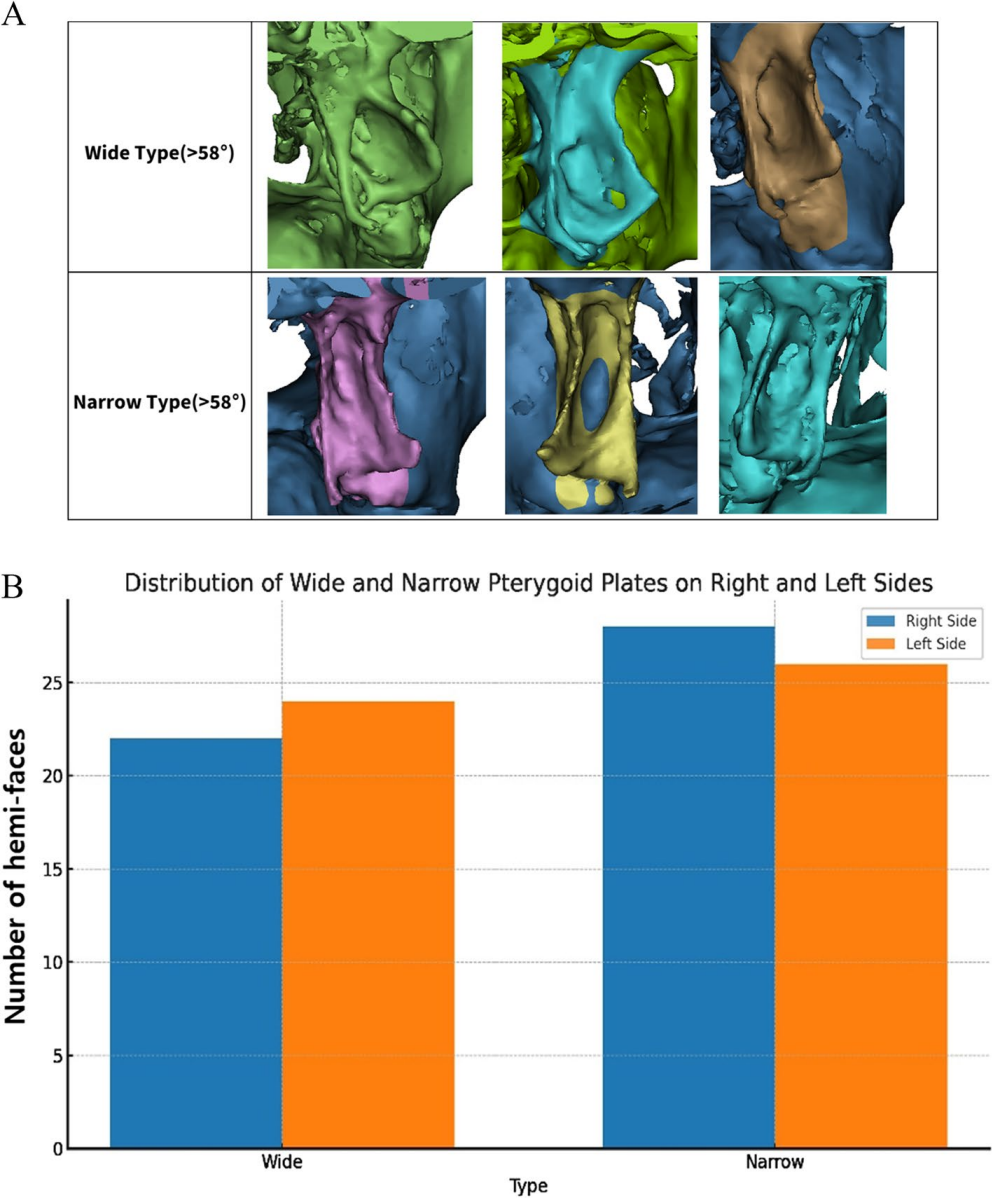
Morphological classification of pterygoid plates based on divergence. **A** Representative images of pterygoid plate by divergence type, **B** Distribution of wide and narrow pterygoid plates on right and left sides

On the right, there were 22 subjects (male: 13, female: 9) with the wide type and 28 subjects (male: 12, female: 16) with the narrow type. On the left, 24 subjects (male: 11, female: 13) had the wide type and 26 subjects (male: 14, female: 12) had the narrow type. There were 20 subjects (male: 11, female: 9) with the wide type on both sides at the same time and 24 subjects (male: 10, female: 14) with the narrow type on both sides at the same time (Table 6, Fig. 7).

**Table 6:** Classification according to the degree of divergence of the pterygoid plate

Pterygoid Plate Morphology	Right Side		Left Side		Bilateral	
	**Male**	**Female**	**Male**	**Female**	**Male**	**Female**
Wide Type	13	9	11	13	11	9
Narrow Type	12	16	14	12	10	14
Total hemi-faces	25	25	25	25	-	-
Total bilateral cases	-	-	-	-	21	23

The "Bilateral" column shows the number of individuals with the same type (wide or narrow) on both sides. Total bilateral cases (21 males + 23 females = 44) represent individuals, not hemi-faces

## Discussion

The purpose of this study was to investigate the functional anatomy of the pterygoid plate and its surrounding structures in the posterior maxilla to provide a better understanding of surgical procedures in this region. In the past, there were studies on various anatomical measurements of the maxilla and skull base. However, most of these previous studies were anatomical studies related to pterygoid dental implants or zygoma dental implants when they were popular [9–13].

While Virtual Surgical Planning (VSP) has become a standard tool in oral and maxillofacial surgery, our study provides several unique benefits that complement and enhance the use of VSP.

First, our comprehensive set of standardized measurements serves as a valuable reference for surgeons, allowing them to quickly compare patient-specific VSP data with population norms. This can help in identifying unusual anatomical variations that might require special consideration during surgery.

Second, our study offers data on anatomical variations across a larger population, which can inform surgeons about the range of normal variations they might encounter. This population-level data is particularly valuable when interpreting individual VSP models, as it provides context for what constitutes a significant deviation from the norm.

Third, our novel classification of pterygoid plate morphology adds a qualitative dimension to the typically quantitative VSP approach. Understanding the prevalence and characteristics of different pterygoid plate types can aid in preoperative planning and intraoperative decision-making, especially in complex cases where the pterygoid plates are involved. For instance, knowing that a patient has a double concave type pterygoid plate might influence the surgeon's approach to pterygoid plate resection during maxillectomy. We also classified the divergence of pterygoid plates as wide or narrow based on a median angle of 58 degrees, with the narrow type being more prevalent in our cohort. This information is crucial for surgical planning, particularly in endoscopic approaches.

Lastly, the detailed 3D measurements and classifications presented in this study have significant educational value. They serve as a comprehensive resource for training residents and fellows in maxillofacial surgery, thereby enhancing their ability to interpret and utilize VSP effectively. By providing a thorough understanding of the complex 3D anatomy of the pterygoid region, our study can help surgeons-in-training to better appreciate the nuances of VSP models and make more informed decisions during surgery.

In the context of endoscopic approaches, such as the endoscopic transpterygoid approach, our detailed measurements of the pterygoid plate at different levels (upper, middle, and lower) provide crucial information that complements VSP. While VSP offers patient-specific models, our population-level data on these measurements can help surgeons anticipate the range of variations they might encounter, even in areas that may not be fully visible during the endoscopic procedure.

Our study methodology represents a significant advancement over previous research. Using skulls of 142 Koreans, Ryu (2016) has investigated the incidence of pterygospinous/pterygoalar bars [14]. Youssef (2014) has performed functional anatomy measurements using CT images of 28 people (14 males and 14 females) for anatomical studies related to the endonasal endoscopic transpterygoid approach [15]. Lee (2001) has conducted an anatomical study of the pyramidal process of the palatine bone using 54 Korean skulls [16]. Most studies were conducted using a cadaver dry skull and a caliper or were measured on 2D CT images [16]. In contrast, our study used 3D reconstruction software (Mimics® 20.0, 3-Matic® 12.0, Materialise, Leuven, Belgium), allowing for accurate measurements even in areas where physical measurement is challenging. The accuracy of repeated measurements was also measured using Dahlberg’s error and RME. The ICC also showed a high reliability of over 0.82 for all items.

We introduced several new anatomical measurements, including dimensions at various levels of the pterygoid plate, pterygoid junction to foramen ovale distance, lateral pterygoid plate area, and divergence of pterygoid plates. These measurements, previously unpublished, provide crucial information for surgeons operating in this complex region.

Our findings align with and expand upon previous research. For instance, in a study related to endoscopic skull base dissection, the distance between the posterosuperior boundary of pterygopalatine fossa and the anteromost border of the foramen ovale was measured and the average distance was 17.1 mm [17], almost identical to our finding of 17.62 ± 2.4 mm. In that previous study, only the length of the pterygoid plate in contact with the skull base was measured. However, in our study, lengths of middle and inferior portions were measured to construct the dimension library necessary for resection of the pterygoid plate according to the tumor level. Uchida et al. reported a mean distance between the lowest posterior point of the maxillary tuberosity to the most lateral lowest point of the pterygomaxillary fissure of 18.7 ± 3.9 mm [18], comparable to our data (Pt.Jn-inf.PPF) of 18.95 ± 2.58 mm on the right and 19.16 ± 2.40 mm on the left. We also introduced the Zy measurement point, defined as the most concave point of the maxillary-zygomatic buttress area. This is crucial for total or subtotal maxillectomy procedures, especially when using a transoral approach with limited visibility.

During maxillectomy and the endoscopic transpterygoid approach, pterygoid plate resection is necessary and pterygoid plate resection must be performed appropriately at the upper/middle/lower positions of the pterygoid plate depending on the location of the tumor in the maxilla and maxillary sinus [7]. Dimensions according to the vertical position of the pterygoid plate have never been reported.

When performing transverse resection of the pterygoid plate after an endoscopic transpterygoid approach or Le Fort I osteotomy, the length and angle of the pterygoid plate bony structure behind the posterior wall of the maxillary sinus are not visible from the surgeon's perspective. If the resection is not precise, intraoperative complications such as brisk bleeding may occur due to damage to the surrounding soft tissue. However, if the surgeon has this information before performing pterygoid plate resection, the surgeon can perform the surgery more safely.

The shape of the pterygoid plate varies greatly from person to person. Few related studies exist. A 2020 study by Sritara et al. reported muscle insertion on the lateral surface of the pterygoid plate and the medial surface of the condyle of the mandible [19]. In 2011, Orhan et al. reported results of analyzing the morphology of the pterygoid Hamulus using CBCT [20]. In 2014, An et al. classified and reported the fracture pattern of the pterygoid process in 100 maxillary trauma patients [21].

In the present study, the lateral pterygoid plate was morphologically classified as middle convex, double concave, flattened, or middle concave. The most common type, including both left and right sides, was the middle convex type.

This study involved anatomical measurements of the posterior maxilla and pterygoid plate area. There were no statistically significant differences in any measurements between left and right sides. With regard to differences between sexes, a previous study showed no statistically significant difference between sexes [18]. However, this study revealed statistically significant differences in the pterygoid height(left), Zy-IOF(right), lateral pterygoid area(left) between sexes. The pterygoid height was significantly longer in men than in women (left). Additionally, Zy-IOF was greater in men than in women (right). Surface area of lateral pterygoid plate was significantly greater in men than in women (left). These results can help us apply relevant information during surgery for men and women. Distances of Zy-IOF (right) and pterygoid height (left) are shorter in women than in men. Thus, care must be taken to avoid invading major surrounding anatomical structures.

Lateral and medial pterygoid muscles are attached to the pterygoid plates and inserted into the mandible. They are important muscles that can affect opening and closing movements of the mandible. Many studies have shown that temporomandibular disorders such as anterior disc displacement are related to dysfunction of the lateral pterygoid muscle [22, 23]. However, there have been no studies on the area of the lateral pterygoid plate to which the lateral pterygoid muscle attaches. This is because there is practically no method for quantitatively measuring the surface of the lateral pterygoid plate. Most measurement studies have been limited to measuring the length, angle, and area between two points within the 2D plane of the DICOM image taken by CT. However, since the pterygoid plate is composed of a surface with a curvature in 3D space, it cannot be measured.

In this study, after reconstructing the CT DICOM file into a 3D model, the lateral surface of the lateral pterygoid plate was separated and the area was further divided into areas of numerous small triangles. By doing so, it was possible to measure the area by calculating the area. While the immediate clinical application of this measurement might not be apparent, we believe it contributes significantly to a comprehensive understanding of pterygoid plate anatomy and could be valuable for future research and clinical applications. Particularly, we see potential relevance to temporomandibular disorders (TMD), especially in cases of anterior-medial disc displacement. The surface area of the lateral pterygoid plate, being the attachment site for the lateral pterygoid muscle, could provide valuable insights into the biomechanics of the temporomandibular joint. Variations in the surface area might correlate with the muscle's potential force generation or its tendency for hyperactivity, which has been implicated in certain types of TMD [24, 25].

Future studies could investigate potential correlations between the surface area of the lateral pterygoid plate and the incidence or severity of TMD. Additionally, this measurement could inform biomechanical models of the temporomandibular joint, potentially leading to improved understanding of TMD pathophysiology and more targeted treatment approaches.

Although we put much effort into this study, there are also limitations to this study. First, in the process of reconstructing DICOM data into a 3D model, the bone density is different for each person. Some errors might have occurred in the process of setting the threshold. Second, since measurements were made with software using a 3D model of skull data, some artificial errors might have occurred. Third, because the patient group was randomly selected, it was not a completely normal population. There might have been changes in anatomical measurements due to patient's existing disease.

Additionally, we acknowledge that we did not explicitly check for three-dimensional congruence between the left and right sides of the surface models. While our study provides detailed measurements and comparisons between left and right sides, a comprehensive analysis of 3D congruence could potentially reveal subtler asymmetries or patterns not captured by our current methodology. This limitation presents an opportunity for future research, where advanced 3D mapping and congruence analysis techniques could be employed to further our understanding of bilateral symmetry and variations in pterygoid plate morphology. Such analysis could provide valuable insights into developmental patterns and potential implications for surgical planning in cases where symmetry (or lack thereof) is clinically significant.

Further research should be conducted in the future to measure the accuracy of resection during pterygoid plate resection using data of corresponding measurements.

In conclusion, while VSP provides patient-specific data, our study offers a broader context that can enhance the interpretation and application of VSP in clinical practice. The combination of VSP with the population-level data, standardized measurements, and morphological classifications provided by our study can lead to more informed surgical planning and potentially improved outcomes in maxillofacial surgery.

## Conclusions

The newly presented functional anatomical knowledge between the pterygoid plate and the anatomical structures of the posterior and superior parts of the maxilla and skull base will be useful to surgeons. This is helpful information for both traditional open surgery and endoscopic surgery. It is believed that functional anatomical data related to surgery of the maxillary sinus, skull base, and infratemporal fossa areas will be of great help to maxillofacial surgeons, head and neck surgeons, and skull base neurosurgeons as they can reduce the risk of damage to major anatomical structures during surgery.

## Supplementary Information

Supplementary Material 1.

## Data Availability

The datasets used and/or analyzed during the current study are available from the corresponding author on reasonable request.

## Abbreviations

Zy: Zygomatic buttress most concave point
IOF: Infraorbital fissure
ANS: Anterior nasal spine
PNS: Posterior nasal spine
N: Nasion
AP: Antero-posterior
MSLM: Maxillary sinus length, medial
MSLL: Maxillary sinus length, lateral
AMA: Anterior maxillary wall angulation
ME: Measurement error
RME: Relative measurement error
SD: Standard deviation
LoA: Limit of agreement
ICC: Intraclass coefficient
mPt: Medial pterygoid plate
lPt: Lateral pterygoid plate
Or: Orbitale
DICOM: Digital Imaging and Communications in Medicine
Pt. Jn.: Pterygomaxillary Junction
Zy: Zygomatico-maxillary buttress point
ICA_mPt: Internal Carotid Artery to Medial Pterygoid Plate
ICA_lPt: Internal Carotid Artery to Lateral Pterygoid Plate
CT: Computed Tomography
CBCT: Cone Beam Computed Tomography
VSP: Virtual Surgical Planning
