# Supplementary material for: Three-dimensional digital anatomical measurements of pterygoid plates and posterior maxillary region
Source: BMC Oral Health. 2024 Dec 18;24:1494. doi: 10.1186/s12903-024-05176-8 (PMC11656809; doi:10.1186/s12903-024-05176-8)
Supplement: Supplementary file 1 — Supplementary Material 1. [file 12903_2024_5176_MOESM1_ESM.docx]

### **Supplementary Table S1: 95% Confidence Intervals for Parameters in Table 4**

| **Parameter** | **Side** | **Mean ± SD** | **95% CI** | **Mean ± SD** | **95% CI** |
| --- | --- | --- | --- | --- | --- |
|  |  | **Male** | | **Female** | |
| **Pterygoid Superior** | Right | 17.82 ± 2.73 | (16.69, 18.95) | 17.41 ± 2.15 | (16.52, 18.30) |
|  | Left | 17.69 ± 3.01 | (16.45, 18.93) | 17.71 ± 1.90 | (16.92, 18.50) |
| **Pterygoid Middle** | Right | 13.67 ± 3.25 | (12.32, 15.02) | 13.09 ± 3.55 | (11.61, 14.57) |
|  | Left | 13.49 ± 3.57 | (12.00, 14.98) | 12.60 ± 3.21 | (11.27, 13.93) |
| **Pterygoid Inferior** | Right | 14.10 ± 4.33 | (12.29, 15.91) | 13.03 ± 3.72 | (11.47, 14.59) |
|  | Left | 15.11 ± 4.38 | (13.28, 16.94) | 13.01 ± 3.78 | (11.42, 14.60) |
| **Divergence (°)** | Right | 59.81 ± 12.02 | (54.93, 64.69) | 57.48 ± 13.33 | (52.05, 62.91) |
|  | Left | 58.68 ± 11.76 | (53.91, 63.45) | 57.30 ± 12.90 | (52.04, 62.56) |
| **MSLM (mm)** | Right | 38.87 ± 3.40 | (37.46, 40.28) | 37.00 ± 3.88 | (35.39, 38.61) |
|  | Left | 39.93 ± 3.28 | (38.57, 41.29) | 37.14 ± 4.91 | (35.08, 39.20) |
| **MSLL (mm)** | Right | 34.27 ± 4.28 | (32.47, 36.07) | 32.20 ± 4.12 | (30.47, 33.93) |
|  | Left | 33.40 ± 4.09 | (31.68, 35.12) | 31.62 ± 4.43 | (29.75, 33.49) |
| **AMA (°)** | Right | 66.19 ± 7.40 | (63.05, 69.33) | 62.85 ± 8.68 | (59.17, 66.53) |
|  | Left | 66.51 ± 8.04 | (63.10, 69.92) | 61.77 ± 7.20 | (58.72, 64.82) |
| **ICA_mPt (mm)** | Right | 29.32 ± 2.87 | (28.12, 30.52) | 27.41 ± 3.86 | (25.81, 29.01) |
|  | Left | 28.45 ± 3.11 | (27.15, 29.75) | 27.38 ± 3.14 | (26.07, 28.69) |
| **ICA_lPt (mm)** | Right | 21.21 ± 5.51 | (18.91, 23.51) | 20.48 ± 4.17 | (18.73, 22.23) |
|  | Left | 20.90 ± 5.27 | (18.70, 23.10) | 20.80 ± 4.46 | (18.93, 22.67) |
| **Or_Ovale (mm)** | Right | 57.76 ± 3.66 | (56.25, 59.27) | 56.37 ± 4.06 | (54.66, 58.08) |
|  | Left | 57.47 ± 3.36 | (56.08, 58.86) | 56.25 ± 3.90 | (54.61, 57.89) |
| **Pt. Jn. inf PPF (mm)** | Right | 18.60 ± 2.87 | (17.40, 19.80) | 19.31 ± 2.25 | (18.37, 20.25) |
|  | Left | 19.28 ± 2.53 | (18.22, 20.34) | 19.03 ± 2.30 | (18.07, 19.99) |
| **Pterygoid height (mm)** | Right | 29.33 ± 2.52 | (28.28, 30.38) | 27.66 ± 2.02 | (26.82, 28.50) |
|  | Left | 29.76 ± 2.99 | (28.51, 31.01) | 27.75 ± 1.88 | (26.97, 28.53) |
| **Pt. Jn. Ovale (mm)** | Right | 31.44 ± 2.28 | (30.49, 32.39) | 30.28 ± 1.94 | (29.47, 31.09) |
|  | Left | 31.92 ± 2.66 | (30.82, 33.02) | 29.65 ± 4.28 | (27.86, 31.44) |
| **Zy_IOF (mm)** | Right | 27.26 ± 1.86 | (26.48, 28.04) | 25.94 ± 1.58 | (25.28, 26.60) |
|  | Left | 27.57 ± 1.92 | (26.77, 28.37) | 25.98 ± 1.68 | (25.28, 26.68) |
| **Lateral pterygoid area (mm²)** | Right | 525.68 ± 86.06 | (489.90, 561.46) | 462.63 ± 111.58 | (416.62, 508.64) |
|  | Left | 531.56 ± 72.81 | (501.48, 561.64) | 455.65 ± 90.39 | (418.13, 493.17) |
